# Direct Relationship Between Heparin Binding to Midkine and Pleiotrophin and the Development of Acute Deep Vein Thrombosis

**DOI:** 10.3390/biomedicines14010242

**Published:** 2026-01-21

**Authors:** Suna Aydin, İsmail Polat, Kevser Tural, Nurullah Duger, Kader Ugur, İbrahim Sahin, Suleyman Aydin, Do-Youn Lee

**Affiliations:** 1 Department of Cardiovascular Surgery, Fethi Sekin City Hospital, Elazig 23300, Türkiye; cerrah52@hotmail.com; 2 Department of Cardiology, Fethi Sekin City Hospital, Elazig 23300, Türkiye; drismailpolat49@gmail.com; 3 Department of Cardiovascular Surgery, Mehmet Akif Ersoy Thoracic and Cardiovascular and Surgical Training and Research Hospital, İstanbul 34303, Türkiye; ktrl2011@hotmail.com; 4 Department of Periodontology, Faculty of Dentistry, Firat University, Elazig 23119, Türkiye; nduger@firat.edu.tr; 5 Department of Internal Medicine (Endocrinology and Metabolism Diseases), School of Medicine, Firat University, Elazig 23119, Türkiye; kaderaksoy06@hotmail.com; 6 Firat Hormones Research Group, Department of Medical Biochemistry and Clinical Biochemistry, School of Medicine, Firat University, Elazig 23119, Türkiye; ibrahimsahin@erzincan.edu.tr; 7 Department of Medical Biology, School of Medicine, Erzincan Binali Yildirim University, Erzincan 24100, Türkiye; 8 College of General Education, Kookmin University, Seoul 02707, Republic of Korea

**Keywords:** deep vein thrombosis, heparin, heparin-binding, midkine, pleiotrophin, low-molecular-weight heparin

## Abstract

**Background/Objectives**: The underlying molecular mechanisms of deep vein thrombosis (DVT), which continues to be a major global public health concern, remain unclear. A key component of anticoagulant therapy, heparin (HP) interacts with heparin-binding growth factors including pleiotrophin (PTN) and midkine (MK), both of which have basic amino acid-rich domains that have a strong affinity for HP. The purpose of this study was to determine if changes in the levels of circulating HP, MK, and PTN are linked to the onset of acute DVT. **Methods**: Thirty patients diagnosed with acute DVT by venous Doppler ultrasonography (VDU) and 28 healthy controls with normal VDU findings were enrolled. Serum HP, MK, and PTN concentrations were measured using ELISA. In DVT patients, blood samples were obtained before and after routine subcutaneous low-molecular-weight heparin treatment; controls provided a single blood sample. ROC curve analysis was used to assess diagnostic performance. **Results**: Prior to treatment, patients with acute DVT exhibited significantly lower serum HP levels (*p* < 0.05) and significantly higher MK and PTN levels compared with healthy controls (both *p* < 0.05). Following heparin administration, serum HP levels increased significantly (*p* < 0.05), while MK and PTN levels showed a decreasing trend that did not reach statistical significance (*p* > 0.05). ROC curve analysis demonstrated limited diagnostic performance for HP (sensitivity 10.3%, specificity 68.8%), PTN (62.1%, 54.2%), and MK (82.8%, 35.4%). **Conclusions**: Decreased circulating HP and increased MK and PTN levels are characteristics of acute DVT that may indicate endogenous HP sequestration through binding to these growth factors. This imbalance could lead to less free HP being available, which would encourage the formation of thrombus. Therapeutic approaches that target MK- and PTN-mediated HP interactions may constitute a unique approach for the therapy of acute DVT, as evidenced by the partial normalization seen after exogenous heparin delivery.

## 1. Introduction

A significant public health issue, deep vein thrombosis (DVT) affects roughly 1–2 people out of every 1000 each year [1]. Geographical location, age, and related risk factors all affect its frequency [2,3]. Higher prevalence has been shown in industrialized nations, especially in Northern Europe, the US, and Australia [4]. DVT is a thrombotic disorder resulting from impaired venous blood flow, most commonly in the lower extremities, and may cause venous obstruction with potentially life-threatening complications, including pulmonary embolism [5].

Clinically, DVT can result in symptoms that appear suddenly and resemble those of a pulmonary embolism (PE) [6]. The condition is more common in men and becomes more common as people age [3]. The conventional explanation for the pathophysiology of DVT is Virchow’s triad: endothelial damage, venous stasis, and hypercoagulability [7]. These interconnected pathways lead to acute deep vein thrombosis (ADVT), particularly in the presence of risk factors such as prolonged immobility, surgery, trauma, malignancy, hormonal therapy, and hereditary predisposition [8,9]. The molecular mechanisms driving DVT are still not fully understood, despite advancements in diagnosis and treatment.

Heparin (HP), a highly sulfated glycosaminoglycan (GAG) discovered in 1916 by Jay McLean and William Henry Howell [10,11], has been a cornerstone of anticoagulant therapy since its first presentation in 1935 [12,13]. Because of its strong anticoagulant properties, unfractionated heparin is frequently used in the prevention and treatment of DVT, pulmonary embolism, and atrial fibrillation [14,15]. A key component of contemporary DVT treatment is low-molecular-weight heparin (LMWH) [15]. HP is frequently utilized for thromboprophylaxis during cardiac surgery, and extracorporeal circulation in addition to its therapeutic uses [16,17,18]. Despite the fact that HP is naturally produced by mast cells and basophils in organs such the liver, lungs, and vascular system, hypercoagulable disorders are frequently observed in clinical practice [19,20]. It is yet unknown what mechanisms underlie this discrepancy.

Midkine (MK) and pleiotrophin (PTN) are members of the heparin-binding growth factor family, according to recent research [21,22,23]. MK is produced physiologically in the bronchial epithelium, lymphocytes, and epidermis and is highly expressed during embryogenesis [24,25]. It is a highly basic, non-glycosylated cytokine with a molecular weight of about 13.3 kDa and 121 amino acids in humans [23,26]. PTN is an 18 kDa growth factor that is largely conserved throughout species and has a great affinity for heparin [21,22,27,28,29]. MK and PTN have overlapping pleiotropic functions, such as roles in angiogenesis and endothelial control, and have about 50% sequence similarity [30,31].

Interactions between HP, MK, and PTN may alter the coagulation and inflammatory pathways implicated in DVT due to their significant affinity for heparin. Hypercoagulability may result from elevated circulating levels of MK and PTN interfering with HP’s anticoagulant effect. The clinical relevance of these changes is still unknown, though. Thus, the purpose of this study is to measure the levels of circulating HP, MK, and PTN in ADVT patients using enzyme-linked immunosorbent assay (ELISA) in order to shed light on their involvement in the pathophysiology of DVT and to support better diagnostic and preventive measures.

## 2. Materials and Methods

The current study was approved by the Fırat University Non-invasive Procedures Ethics Committee on 19 April 2018, with issue number 08 and decision number 13. This prospective case–control study was conducted at Fethi Sekin City Hospital between 19 April 2018 and 1 October 2023, adhering to the ethical standards set forth in the previous and latest versions of the Declaration of Helsinki (2024 revision) [32]. Participants were informed about the study, and their informed consent was obtained.

### 2.1. Study Design

Each participant in this study was randomly selected. Thirty patients (16 males and 14 females, age range: 55 ± 11 years) diagnosed with acute DVT after venous Doppler ultrasonography and healthy volunteers with no vascular disease and normal venous Doppler ultrasonography findings [33] who came to our hospital for annual check-ups were included in the study. The control group (*n*: 28; 16 males and 12 females, age range: 52 ± 9 years) were also evaluated according to Wells DVT criteria (those with a Wells DVT score below 2 were included in the study) [34]. In addition, detailed anamnesis was taken from all cases and subcutaneous LMWH was administered for routine treatment [35].

The exclusion criteria for this clinical study were as follows [33]: those with a previous DVT attack, those with active cancer, those with diabetes mellitus (regardless of type 1 and 2), those with obesity (body mass index > 30 kg/m^2^), those with a history of myocardial infarction within the last month, those with heart failure, pulmonary embolism, acute coronary syndrome, kidney or liver dysfunction, those with cardiovascular risk factors [hypotension and hypertension, hyperlipidemia (>2.5 g/L), those <21 and >65 years of age (geriatric patients), and those using intravenous drugs. Patients with a positive family history, pregnancy, those receiving warfarin therapy and those undergoing immobilization (bed rest, general anesthesia, surgeries or surgical interventions, long flights), those with peripherally inserted venous catheters, those with vasculitis or varicose veins, those with any known systemic disease (systemic lupus erythematosus, inflammatory bowel disease, and nephrotic syndrome), those with burns, and those using oral estrogen were excluded from the study.

### 2.2. Collection and Storage of Biological Samples

Sample collection and storage were performed in accordance with previously described procedures [36]. In total, 5 mL of blood was collected from patients before and after treatment (follow-up blood was collected on day 8), and 5 mL of blood was collected from control participants in biochemistry tubes. These blood samples were centrifuged at 4000 rpm (1792 g force) for 5 min. The resulting sera were stored in Eppendorf tubes at −80 °C until use [36].

Information on the manufacturers and molecules of the kits used in ELISA analyses: HP: Human HP ELISA Kit (Shanghai Sunredbio Technology Co., Ltd., Shanghai, China, Catalog No: 201-12-1130); sensitivity: 0.05 ng/mL, detection range: 0.05–15 ng/mL. MK: Human MK ELISA Kit (Shanghai Sunredbio Technology Co., Ltd., Shanghai, China, Catalog No: 201-12-7339); sensitivity: 4.006 ng/L, detection range: 5–1500 ng/L. PTN: Human PTN ELISA Kit (Shanghai Sunredbio Technology Co., Ltd., Shanghai, China, Catalog No: 201-12-0163); sensitivity: 7.118 ng/mL, detection range: 8–2200 ng/mL. The manufacturer’s catalog specifies the measurement accuracy, and the intra-assay coefficients of variation (CV) for all ELISA kits used were below 10%, while inter-assay CVs were below 12%, indicating acceptable analytical reproducibility and precision. Also, in this study, we validated the assay to determine the accuracy with which low molecular weight heparin (LMWH) is measured, following the method previously described by Aydın et al. [36]. The intra-assay coefficients of variation were below 10%, and the inter-assay CVs were below 15%, indicating that the kit measures LMWH with a sensitivity comparable to that of endogenous heparin.

### 2.3. Analysis of HP, MK, and PTN Molecules Using ELISA

The ELISA method is a frequently used immunological test in scientific research [36]. It is a measurement method based on the demonstration of color changes resulting from the use of an antigen and an enzyme-linked conjugate, along with the reaction of antibodies to the antigen [36]. These molecules were studied using the ELISA method according to the instructions provided in the manufacturer’s catalog and previously published guidelines. The steps in this method are presented briefly as follows: (1) bringing all reagents to room temperature and performing the analysis at room temperature. (2) Preparing standard solutions using the procedure specified in the kit catalog and determining the number of wells required for analysis. (3) Adding the standard solution in the amount specified by the company to the wells where the standard will be placed. (4) Adding the serum sample specified in the catalog to the sample wells. (5) Adding the biotinylated antibody in the amount specified in the catalog to the sample wells. (Note: Since the standard solution contains biotinylated antibodies, antibodies should not be added to the standard wells). (6) Add streptavidin-HRP in the amount specified in the catalog to all wells except the blank well. (7) Cover the microplate and incubate at 37 °C for 60 min. (8) After incubation, wash the microplate 5 times with the prepared wash buffer and dry the plate with a paper towel or other absorbent material after washing. (9) Add Chromogen A and Chromogen B substrate solutions in the amount specified in the catalog to all wells. (10) Cover the microplate and incubate at 37 °C for 10 min. (11) Add stop solution in the amount specified in the catalog to all wells (blue colors turned yellow). (12) Read the absorbances at 450 nm on an ELISA microplate reader within 10 min after adding the stop solution and determining the concentrations corresponding to these absorbances using a ChroMate microplate reader (Awareness Technology Inc., Palm City, FL, USA). Throughout this study, a Bio-Tek ELX50 automatic washer (BioTek Instruments, Winooski, VT, USA) was used for washing procedures [36].

### 2.4. Statistical Analysis

SPSS (Statistical Package for the Social Sciences) version 25 (SPSS Inc., Chicago, IL, USA) was used for all statistical analyses. The Shapiro-Wilk test was used to examine the measured values’ normality distribution. When comparing parameters between several related groups, the Wilcoxon rank test was employed; when comparing parameters between unrelated groups, the Mann-Whitney U was utilized. The Spearman correlation test was used to evaluate potential relationships between the variables under analysis. All statistical results are expressed as the mean ± standard deviation, rounded to two decimal places. A *p*-value of <0.05 was considered statistically significant. The best cut-off level for HP, MK, and PTN prior to treatment as well as their sensitivity and specificity for DVT were ascertained using receiver operating characteristic (ROC) curve analysis. Additionally, the diagnostic efficacy of HP, MK, and PTN for DVT was assessed using the area under the ROC curve (AUC).

## 3. Results

The ages and body mass indexes of the participants were similar, with no statistically significant difference (>0.05; Table 1). Furthermore, no significant differences were found when comparing some biochemical parameters between the groups (>0.05, Table 1). When we examined the distribution of DVT involvement by anatomical region, from most to least, it was found to be femoral, popliteal, iliac, and limb (Table 2).

**Figure 1:**
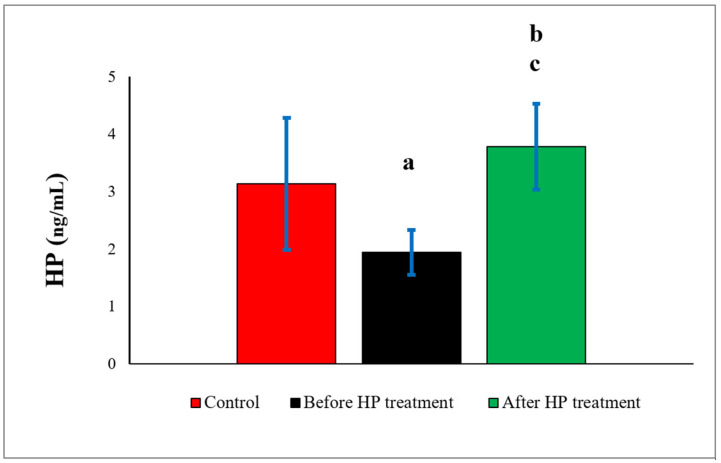
Comparison of HP values between controls and DVT patients before and after HP treatment. HP: heparin. a: control group versus before HP treatment group (*p* = 0.001). b: control group versus after HP treatment group (*p* = 0.024). c: before HP treatment group versus after HP treatment group (*p* = 0.000).

When HP amounts were compared with the control group (3.13 ± 1.15), a statistically significant decrease (1.94 ± 0.39) (*p* = 0.001) was found in the pre-treatment group, and also statistically significant increase (3.78 ± 0.75) (*p* = 0.024) was found in the post-treatment group. When comparing the pre-treatment and post-treatment groups, the difference was statistically significant (*p* = 0.000) (Figure 1).

**Figure 2:**
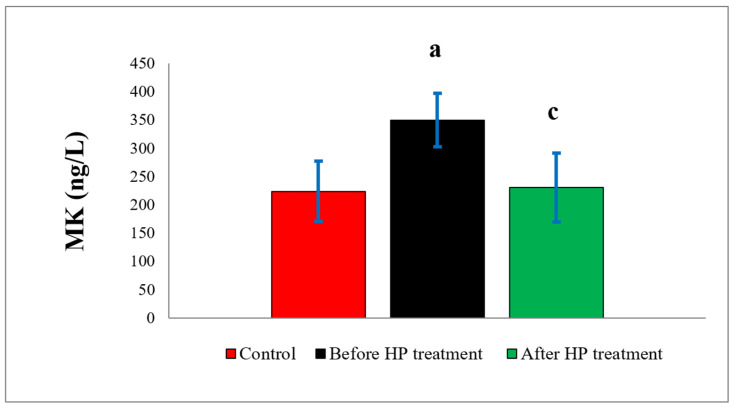
Comparison of MK values between controls and DVT patients before and after HP treatment. MK: midkine; HP: heparin. a: control group versus before HP treatment group (*p* = 0.000). c: before HP treatment group versus after HP treatment group (*p* = 0.000).

MK levels were found to be higher in the treatment groups compared to the control group (223.73 ± 53.09). The increases were statistically significant (*p* = 0.000) in the pre-treatment (349.60 ± 47.09) group and insignificant (*p* = 0.648) in the post-treatment (230.50 ± 61.06) group. Compared to the pre-treatment group, MK levels were found to decrease statistically significantly (*p* = 0.000) in the post-treatment group (Figure 2).

**Figure 3:**
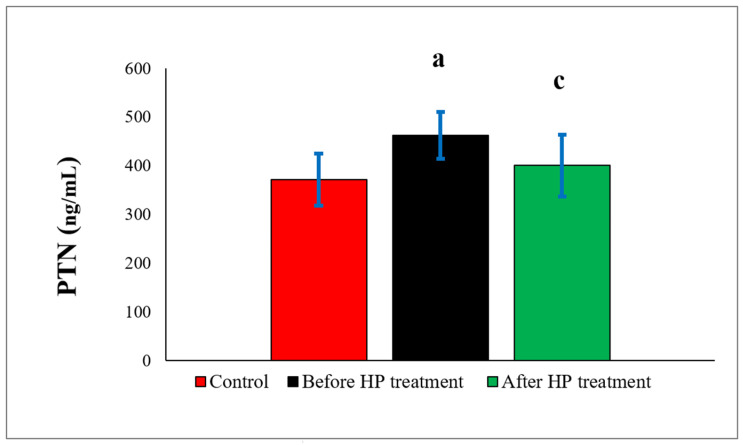
Comparison of PTN values between controls and DVT patients before and after HP treatment. PTN: pleiotrophin; HP: heparin. a: control group versus before HP treatment group (*p* = 0.046). c: before HP group versus after HP treatment group (*p* = 0.038).

PTN levels were found to be higher in both the pre-treatment (462.64 ± 48.09) and post-treatment (401.01 ± 63.54) groups compared to the control (371.75 ± 53.15) group. The increases were statistically significant (*p* = 0.046) in the pre-treatment group and insignificant (*p* = 0.645) in the post-treatment group. When the treatment groups were compared among themselves, the difference was found to be statistically significant (*p* = 0.038) (Figure 3).

**Figure 4:**
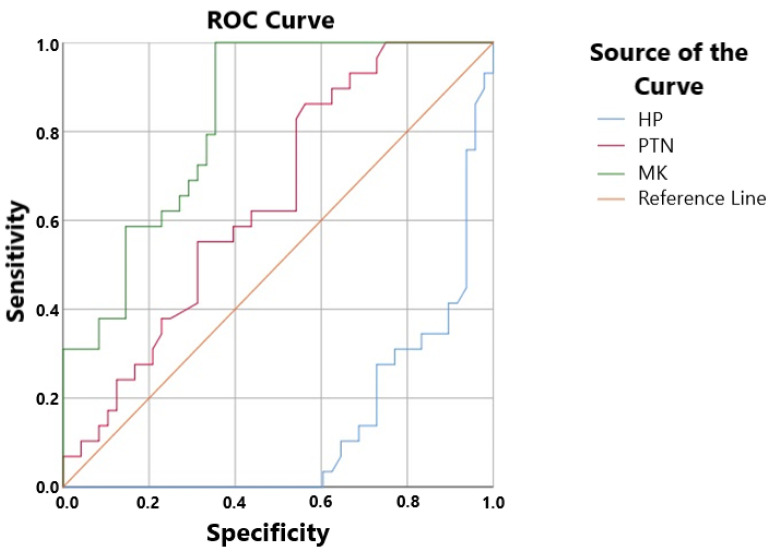
HP, PTN, and MK levels are analyzed using a receiver operating characteristic curve (RO). MK stands for midkine, PTN for pleiotrophin, and HP for heparin. ROC curve analysis of HP, MK, and PTN for acute DVT, showing 4ut-off values of 2.505 ng/mL (HP), 245.185 ng/L (MK), and 361.015 ng/mL (PTN), with corresponding sensitivity and specificity rates.

ROC analysis revealed that the sensitivity and specificity rates for HP levels were 10.3% and 68.8%, respectively. In the pre-treatment group, the HP cut-off value was found to be 2.505 ng/mL. In the pre-treatment group, the cut-off level for MK levels was 245.185 ng/L, the sensitivity rate was 82.8%, and the specificity rate was 35.4% (Figure 4). The ROC studies for PTN yielded a cut-off level of 361.015 ng/mL, a sensitivity rate of 62.1%, and a specificity rate of 54.2%. The correlation analysis results are presented in a table (Table 3).

## 4. Discussion

Patients with acute DVT in the current investigation showed markedly lower circulating heparin (HP) levels together with higher amounts of midkine (MK) and pleiotrophin (PTN), indicating a possible imbalance in endogenous anticoagulants. These findings raise significant questions about how thrombosis develops despite the presence of HP in the circulation, as HP is a naturally occurring anticoagulant produced in the human body. Increased amounts of heparin-binding proteins like MK and PTN may sequester circulating HP, which would decrease the amount of free, physiologically active heparin available and encourage a hypercoagulable condition.

This process might be an underappreciated pathway involved in the pathophysiology of DVT. In addition to these molecular results, our research showed that the femoral vein was most commonly affected by DVT, followed by the popliteal, iliac, and tibialis posterior veins. Our findings are in line with reports that the majority of proximal DVTs affect the femoral and popliteal veins, with iliofemoral involvement accounting for a smaller proportion of cases [37], although some studies have reported higher involvement of the iliac vein compared to the popliteal vein [33]. Our cohort’s representativeness is supported by the anatomical findings’ agreement with previous research, which also raises the possibility that the biochemical changes we saw may be especially significant in proximal DVT. Together, these results lend credence to the theory that, even in the presence of endogenous anticoagulant mechanisms, interactions between HP and heparin-binding growth factors like MK and PTN may contribute to localized hypercoagulability and thrombus formation.

The reported distribution of DVT involvement by anatomical region is explained as follows. The femoral vein is the first major deep vein to receive blood from the legs, is more susceptible to external compression, and, particularly in positions such as sitting, squatting, or lying for long periods, muscles can exert greater pressure on the femoral vein, slowing blood flow and causing stasis, which may be the reason for the highest reported thrombus rate in this anatomical region [38].

The popliteal vein is located within the deep venous system and is one of the points where blood is most prone to pooling, and venous stasis is common, thus increasing the risk of thrombus formation. This may be the reason why thrombus was reported second most frequently in the popliteal vein in this study, after the femoral vein [39]. The iliac vein is located in a more protected anatomical location and is less exposed to external pressure [40]. This may be the reason why thrombus rates were reported lower in the iliac vein compared to the femoral and popliteal veins. The reason for the lower thrombus involvement in limb veins is their smaller diameter. This is because flow in small-diameter superficial or tibialis posterior veins can be faster, resulting in less stasis [40]. This may also be the reason why the lowest thrombus rates were reported in the tibialis posterior veins in this study. In addition to all the above, chronic diseases that damage the endothelial layer or slow blood flow may increase the risk of thrombosis [41]. Furthermore, endogenous or idiopathic issues with anticoagulant pathways may have contributed to the development of thrombus [42].

For the first time, this study looked into whether possible issues with the HP route (anticoagulation) cause DVT (thrombus). By attaching to and activating circulating antithrombin 3 (ATIII), HP and its derivatives work. The active enzymatic clotting factors (Factor II (thrombin), Factors IX, X, XI, and XII) are inhibited by activated ATIII [43,44].

Recently, MK and PTN proteins have been reported to bind to HP [21,45]. Therefore, the primary focus of this study was whether these molecules, bound to heparin, trigger ADVT. In our study, before the implementation of the routine treatment protocol (before LMWH), blood HP, MK, and PTN values in ADVT cases were compared with those in control cases. In DVT cases, blood HP levels were significantly decreased, while MK and PTN values were significantly increased. MK and PTN share approximately 50% sequence homology and constitute a family of heparin-binding growth factors [46]. Both proteins are conserved across a wide range of vertebrates [21,47]. ADVT may have developed due to increased MK and PTN binding to HP, leading to a decreased ability to prevent clot formation. In other words, our current data suggest that because HP bound to MK and PTN cannot adequately prevent blood clots, blood flow may have slowed, stasis may have occurred, and thrombi may have formed. Inflammation significantly contributes to the development and progression of venous thrombosis [48]. MK and PTN expression is generally increased during inflammation, tissue repair, and neoplastic transformation [47,49,50]. Therefore, increased MK and PTN proteins due to inflammation or other causes may bind HP, leading to the development of ADVT [47].

In this study, blood HP levels were found to be increased in patients with ADVT. Moreover, MK and PTN levels showed a significant decrease following the initial administration of LMWH. The observed elevation in blood HP levels in ADVT cases may be associated with the subcutaneous administration of LMWH. MK and PTN bind to both endogenous HP and exogenously administered LMWH; the resulting increase in free HP levels may contribute to the inhibition of thrombus formation and facilitate thrombus resolution. HP consists of negatively charged sulfated glycosaminoglycans, and MK and PTN bind to this structure via their positively charged regions [21,47]. The structure of MK and PTN, particularly at the C-terminal, contains a region rich in basic amino acids (especially lysine and arginine) that bind to HP. Additionally, the N-terminal domain contains some basic sequences that contribute to HP binding [51,52]. Furthermore, the increase in MK and PTN after treatment may be due to the addition of exogenous LMWH to the total circulating HP pool in addition to endogenous HP. This is because, above a certain threshold, the amount of MK and PTN in circulation is insufficient. Therefore, while MK and PTN levels decrease, HP levels may increase. HP has both anticoagulant (anticoagulant) and anti-inflammatory properties [11,53]. HP can bind proinflammatory cytokines and chemokines such as IL-8, IL-6, and TNF-α, inhibiting their biological activity [53]. As we mentioned above, increased MK and PTN due to inflammation in ADVT may be another reason why their amounts decreased due to the reduction in inflammation by exogenous LMWH.

Receiver operating characteristic (ROC) analysis in the current study demonstrated poor diagnostic performance of circulating heparin (HP), with very low sensitivity (10.3%) at a cut-off value of 2.505 ng/mL despite relatively high specificity (68.8%). Given the dynamic nature of endogenous HP and its interactions with heparin-binding molecules, these findings suggest that reduced HP levels alone are insufficient for the accurate diagnosis of acute DVT. Specifically, high amounts of pleiotrophin (PTN) and midkine (MK) may bind and sequester circulating HP, reducing its diagnostic sensitivity and observable free HP concentrations. PTN, on the other hand, showed low specificity (54.2%) and intermediate sensitivity (62.1%) at a cut-off value of 361.015 ng/mL, suggesting a limited capacity for discrimination when employed as a stand-alone biomarker. At a cut-off value of 245.185 ng/L, MK showed the highest sensitivity (82.8%) among the assessed parameters but poor specificity (35.4%), indicating that although elevated MK levels may be common in acute DVT, they are not unique to the illness. All of these results show that not one of the indicators by itself can accurately diagnose DVT. However, rather than acting as accurate diagnostic indicators, the complimentary patterns seen—low HP sensitivity coupled with elevated MK and PTN levels—support the theory that interactions between HP and heparin-binding growth factors may contribute to the pathophysiology of DVT. These findings underscore the potential benefits of a combined biomarker approach and emphasize the need for more research to elucidate the clinical and mechanistic significance of HP–MK–PTN interactions in thrombotic illness.

### Limitations

There are a number of limitations to this study. In addition to limiting statistical power and generalizability, especially for subgroup and ROC analyses, the relatively small sample size and single-center design also prevented multivariable adjustment for potential confounders including age. Furthermore, a carefully chosen DVT cohort was created by purposefully excluding patients with common comorbidities; as a result, the results should be evaluated within this particular clinical context.

An ELISA-based technique that detects both free and protein-bound fractions was used to quantify circulating HP. These measures might not accurately represent physiologically active free heparin because heparin is mostly endothelial-associated and highly protein-bound. Even though the assay was validated, possible interactions with low molecular weight heparin might have affected the results, so careful interpretation is necessary.

PTN and MK have limited diagnostic specificity for acute DVT since they are non-specific inflammatory markers that can be increased in a variety of clinical situations. Furthermore, it is impossible to rule out variability pertaining to concurrent drugs, sample timing, and pre-analytical conditions.

Lastly, the study did not include direct binding or competition experiments, control for acute-phase inflammation, or evaluation of established coagulation endpoints (e.g., antithrombin III activity, anti-Xa levels, thrombin generation, or D-dimer dynamics), even though the observed associations point to a possible interaction between heparin, midkine, and pleiotrophin. Therefore, it is unable to definitively establish causal and functional inferences relating to anticoagulant action.

## 5. Conclusions

This study offers preliminary evidence that heparin binding mediated by MK and PTN is linked to acute deep vein thrombosis, which may impair heparin’s biological activity. Reduced circulating heparin levels and increased MK and PTN were observed with DVT, indicating that MK and PTN may be involved in heparin sequestration. Combined evaluation of several biomarkers or their ratios may better reflect the intricate molecular interactions underlying thrombus development, even though none of these markers by themselves provide enough diagnostic accuracy. These findings warrant further investigation in larger, well-characterized cohorts and emphasize the potential mechanistic and clinical implications of HP, MK, and PTN in acute DVT.

## Figures and Tables

**Table 1:** Comparison of some anthropometric and biochemical parameters of participants.

Parameters	Control (*n*: 28)	ADVT (*n*: 30)
Age (years)	52 ± 9.2	55 ± 11.3
BMI (kg/m^2^)	24.6 ± 1.7	26.3 ± 2.1
Smoking (female/male)	5/7	6/8
Creatinine (mg/dL)	0.91 ± 0.11	0.89 ± 0.12
Gender (female/male)	12/16	14/16
Glucose (mg/dL)	93 ± 6.1	95 ± 4.8
Urea (mg/dL)	37.4 ± 7.9	39.7 ± 9.3

**Table 2:** The most frequently involved anatomical areas of the human body in ADVT.

Anatomically Frequently Involved Veins	*n*	%
Femoral	30	50
Popliteal	16	26
Iliac	10	17
Tibialis posterior	4	7

**Table 3:** Correlation analysis between heparin (HP), midkine (MK), and pleiotrophin (PTN) levels in DVT patients.

Related Groups	*p* Value	*r* Value
MK (control)–HP (before HP treatment)	* 0.040	+0.405
MK (after HP treatment)–HP (before HP treatment)	* 0.012	+0.504
MK (control)–PTN (control)	* 0.007	+0.539
PTN (after HP treatment)–PTN (before HP treatment)	* 0.039	+0.392

Significant positive correlations were observed between MK and HP before and after treatment, as well as between MK and PTN in controls. PTN levels also showed a significant positive correlation before and after HP treatment. (* *p* < 0.05; *r* = Pearson correlation coefficient).

## Data Availability

The raw data supporting the conclusions of this article will be made available by the authors on request.

## Abbreviations

The following abbreviations are used in this manuscript:

ADVT	Acute Deep Vein Thrombosis
ANOVA	One-way Analysis of Variance
ATIII	Antithrombin 3
AUC	Area Under the ROC Curve
CV	Coefficients of Variation
DVT	Deep Vein Thrombosis
Factor II	Thrombin
GAG	Glycosaminoglycan
HP	Heparin
LMWH	Low Molecular Weight Heparin
MK	Midkine
PE	Pulmonary Embolism
PTN	Pleiotrophin
ROC	Receiver Operating Characteristic
VDU	Venous Doppler Ultrasonography
